# A cross sectional study of organizational factors and their impact on job satisfaction and emotional burnout in a group of Australian nurses: infection control practitioners

**DOI:** 10.1186/s12913-021-06477-2

**Published:** 2021-05-10

**Authors:** Katie Page, Nicholas Graves

**Affiliations:** 1 Centre for Health Economics Research and Evaluation, University of Technology Sydney, Level 12, Building 10, 235 Jones Street, PO Box 123, Broadway, 2007 Ultimo, Australia; 2 Duke-NUS Medical School, National University of Singapore, Singapore, Singapore

**Keywords:** Infection control, Nurses, ICP, Organizational factors, job satisfaction, burnout

## Abstract

**Background:**

Infection control practitioners (ICPs) are a group of specialized nurses fundamental to effective healthcare infection prevention and control initiatives. Relative to other groups of nurses much less is known about their working conditions. Organizational factors may impact ICPs’ levels of job dissatisfaction and emotional job burnout and, subsequently, their quality of practice. We measure a range of organizational factors to document the working conditions of ICPs and show how these are linked to job satisfaction and emotional burnout in a sample of Australian ICPs.

**Methods:**

We conducted a cross sectional study using an online survey. All employed ICPs in 50 of the largest public hospitals in Australia were invited to participate. One hundred and fifty three ICPs completed the survey.

**Results:**

ICPs are moderately to highly satisfied with their job but show high levels of emotional burnout, time pressure and cognitive demands. Low job satisfaction was associated with less job control, low perceived organizational support and poor communication. In contrast, emotional burnout was associated with high time pressure and cognitive demands coupled with poor communication.

**Discussion:**

This study provides new evidence about the organizational context of ICPs in Australia, and about the factors that impact on job satisfaction and emotional burnout. These findings may be used to modify national infection prevention and control programs to suit local organizational contexts. Further research is needed to determine the precise nature of these relationships and the downstream impacts on hospital-wide infection control outcomes.

**Conclusions:**

Organizational context and factors are important to consider when evaluating the impact and implementation of infection control programs.

**Supplementary Information:**

The online version contains supplementary material available at 10.1186/s12913-021-06477-2.

## Background

Infection control practitioners (ICPs) are fundamental to the success of any hospital-based healthcare infection prevention and control initiative [1, 2]. This group of people, the majority of whom are nurses, are responsible for a variety of duties in a healthcare facility linked to the prevention and control of hospital acquired infections (HAIs), including direct infection prevention and control activities as well as surveillance, education, policy, research, communication and administration tasks [1, 2]. However, the working conditions of ICPs in Australia, including the organizational contexts in which they practice, are highly diverse and not well documented [1]. Understanding the organizational factors impacting ICPs and their experience of job satisfaction and burnout is important; the extent to which these individuals are satisfied in their jobs and experience job burnout may be key predictors of the quality of the infection prevention practices they implement and, subsequently, of the rates of HAIs in healthcare facilities.

The experience of job dissatisfaction and job burnout are significant problems for Australian nurses. Studies consistently suggest that nurses in Australia experience moderate to high levels of burnout [3–5]. Rates of job dissatisfaction and burnout among ICPs has not been reported in the literature; however, because of the diversity and complexity of ICPs’ roles [1, 2], it is feasible that ICPs’ experience of job dissatisfaction and burnout is comparable to, if not higher than, that of nurses in general practice settings.

There is a known relationship between nurses’ experience of job dissatisfaction and burnout, and reduced quality of patient care. When nurses experience poor job satisfaction and burnout, quality of care is consistently low [6–8], and patients are more likely to report dissatisfaction with the quality of the care they receive [9]. In the context of infection control there is evidence to suggest that when nurses experience poor job satisfaction and burnout, their compliance with infection control initiatives declines [10]. Specifically, poor job satisfaction and burnout have been associated with higher rates or morbidity and mortality [11]. In the context of infection control there is evidence to suggest that when nurses experience poor job satisfaction and burnout, the rates of HAIs may increase [12]. This may be because nurses’ experience of job dissatisfaction and burnout results in negative coping behaviors among nurses, such as distancing themselves from work [13]. Therefore, understanding their working environment is important to improve retention of qualified staff and to maintain high quality healthcare practices.

There is also a relationship between nurses’ experience of job dissatisfaction and burnout and a variety of organizational factors, particularly POS. The literature suggests that where nurses perceive a supportive organizational culture generally [4, 14–16]– and, specifically, where nurses perceive good interdisciplinary collaboration [6], quality management [17] and high levels of safety [9] in their organization – they are more likely to be satisfied with their work and / or report lower levels of burnout. However, there is little evidence for the impact of organizational factors on stress and burnout among nurses working in infection control and prevention roles specifically, including among ICPs. Furthermore, there a paucity of research which considers organizational factors other than general POS (or closely related concepts), and few studies from the Australian context. It is these gaps that this research aimed, in part, to address.

This study was embedded in a larger evaluation of the Australian National Hand Hygiene Initiative (NHHI). The NHHI is a major patient safety program funded by the Australian Commission for Safety and Quality in Health Care and coordinated by Hand Hygiene Australia. The NHHI aims to standardize hand hygiene practices in Australia, as hand hygiene is recognized as the most effective intervention for preventing HAIs [18]. We undertook a comprehensive evaluation of the efficiency and cost-effectiveness of the NHHI, finding that (a) the program is associated with a statistically significant reduction in the rates of some HAIs, and (b) that the program is cost-effective against an Australian threshold of per life year gained [19, 20]. This study was conducted mid-way through the NHHI evaluation in 2014, in hospitals participating in the NHHI program.

This study had two aims: First, to provide a descriptive overview of the working conditions of ICPs in Australia by measuring a range of important organizational factors. Second, to examine the relationship between these organizational factors and ICPs job satisfaction and emotional burnout.

## Methods

Data were collected using a quantitative survey (see Supplementary file 1 ). The survey was administered online with university-based software, KeySurvey. Participants were sent personalized emails with a link to the survey. Reminder emails were sent at 2 weeks and 1 month after the initial email. The survey instructions informed participants that its purpose was to measure their opinions and attitudes about infection prevention and control practices and the support they receive in their hospital. This research was undertaken with approved ethical clearance by the University and the Hospitals’ Human Research Ethics Committees.

### Participants

All of the ICPs in 50 of the largest public hospitals in Australia were invited to participate in the study. 153 of the 215 people invited completed the survey, giving an overall response rate of 71 %. There was representation from all states and territories. Half of the ICP sample was from hospitals in New South Wales and Victoria (50 %, *n* = 77). Approximately one third of the sample was from hospitals in Queensland, South Australia and Western Australia (38 %, *n* = 59). Ninety-three per cent of the sample was female, reflecting the natural gender differences in the profession seen in other studies involving ICPs in Australia [1]. The ages of the participating ICPs ranged from 26 to 65 years, with a median of 44 years. The ICPs had an average of 22.5 years nursing experience, ranging from 5 to 44 years. All participating IPCs had at least 6 months of work experience in their current hospital; this was deemed necessary to ensure they had the minimum knowledge and experience of their workplace and infection control practices required to answer the survey questions meaningfully.

### Measures

The first part of the survey consisted of demographic and background questions including age, number of years of experience in nursing, number of years working in infection control, staff supervisory responsibilities and involvement in hand hygiene auditing. The second part of the survey asked questions on nine key constructs: two main outcome variables of job satisfaction and emotional burnout, and seven predictor variables of perceived organizational support (POS), communication, support from senior management, time pressure, job control, cognitive demands and safety climate. All of these questions are from existing surveys which are available and free for academic use. The survey can been seen in Supplementary file 1.

### Outcome measures

Two key outcome variables were measured in this study: job satisfaction and emotional burnout.

#### Job satisfaction

Job satisfaction was evaluated using a one-item measure developed by [21]. Participants were asked, ‘How do you feel about your job, all things considered?’ Participants answered this question using a five-point Likert scale, from ‘not at all satisfied’ (1) to ‘extremely satisfied, couldn’t be more satisfied’ (5).

#### Emotional burnout

Emotional burnout was evaluated using a validated one-item measure developed by [22]. Participants were asked to select one statement on a scale of ‘I enjoy my work…’ (1) to ‘I feel completely burned out and often wonder if I can go on…’ (5). This question was designed to assess participants’ self-perceived level of burnout based on symptoms such as stress, exhaustion and frustration, etc. [22]. We thus refer to this construct as emotional burnout. This non-proprietary measure has also been validated against the single item measure of emotional burnout on the widely used and cited Maslauch Burnout Inventory [23, 24].

In addition to these two key outcome variables, seven predictor variables were measured in this study: POS, communication, support from senior management, time pressure, job control, cognitive demands and safety climate.

#### Perceived organizational support (POS)

To measure POS, an 8-item scale was used; this was a shortened version of the full 36-item scale developed by [25]. The scale asked participants to rate eight items on a seven-point Likert scale, from ‘strongly disagree’ (1) to ‘strongly agree’ (7). Items included: ’The hospital really cares about my wellbeing’ and ‘My hospital would forgive an honest mistake on my part’.

#### Communication

Communication about infection prevention and control practices was measured using four items taken from scales developed by [26–28]. The questions asked participants to rate items on a five-point Likert scale, from ‘strongly disagree’ (1) to ‘strongly agree’ (5). Items include: ‘I know the proper channels to direct questions regarding hand hygiene’ and ‘Good communication flow exists down the chain of command regarding hand hygiene’.

#### Support from senior management

Perceived support from senior management was measured using eight items taken from scales developed by [26, 27]. The questions asked participants to rate items on a five-point Likert scale, from ‘strongly disagree’ (1) to ‘strongly agree’ (5). Items include: ‘Senior management has a clear picture of the risk associated with poor hand hygiene’ and ‘My suggestions about hand hygiene would be acted upon if I expressed them to senior management’.

#### Time pressure

To measure time pressure, three questions were taken from a tool developed by [29]. The questions asked participants to rate items on a seven-point Likert scale, from ‘never’ (1) to ‘always’ (7). Items included: ‘I have unachievable deadlines’ and ‘I have to neglect some tasks because I have too much to do’.

#### Job control

To measure job control, three questions were taken from a tool developed by [29]. The questions asked participants to rate items on a seven-point Likert scale, from ‘never’ (1) to ‘always’ (7). Items included: ‘I have a choice in deciding what I do at work’ and ‘I have a choice in how I do my work’.

#### Cognitive demands

Cognitive demand was measured using four questions taken from a tool developed by [30]. The questions asked participants to rate items on a seven-point Likert scale, from ‘never’ (1) to ‘always’ (7). Items include: ‘Do you have to concentrate all the time to watch for things going wrong?’ and ‘Do you have to react quickly to prevent problems arising?’

#### Hospital safety climate

Hospital safety climate was measured using a 16-item questionnaire developed by [31]. The questions asked participants to rate items on a six-point Likert scale, from ‘strongly disagree’ (1) to ‘strongly agree’ (6). Items include: ‘My hospital reacts quickly to solve the problem when told about infection-related risks’ and ‘My hospital tries to continually improve hand hygiene compliance in each ward’.

All scale reliabilities were determined using Cronbach’s statistic. Two separate multiple regression analyses were conducted to predict job satisfaction and emotional burnout. The seven predictor variables: POS, time pressure, job control, communication, hospital level safety climate, cognitive demands, and support from senior management were added to the model simultaneously in one block. Age and years of experience in infection control were entered into the model when they were significant with the outcome variable. Results are presented separately for each outcome measure in the following section. A series of one-way ANOVAs (analysis of variance) were used to test for state/territory differences in all of the measures but there were no notable differences between the states/territories. We used Green’s rule of thumb (medium effect) to test the necessary sample size for the entire model: n = 50 + 8*predictors = 50 + 8*9 = 122 [32]. We included 9 predictors when there are only 7 used in order to allow for the demographic variables. Our sample is therefore an adequate sample size to test the model and the significance of the predictors.

## Results

Table 1 shows the scale reliabilities for the seven organizational factors. All reliabilities were acceptable and met Nunnally’s criterion [33, 34].

**Table 1:** Scale reliabilities for predictor variables

#	Scale	# items	Cronbach’s alpha
1	Perceived organizational support	8	0.93
2	Communication	4	0.82
3	Senior management support	8	0.91
4	Time pressure	3	0.88
5	Job control	3	0.82
6	Cognitive demands	4	0.78
7	Safety climate	16	0.94

Table 2 shows the means and standard deviations for all measures.

**Table 2:** Descriptive statistics

Variable	*N*	Mean	SD
Perceived organizational support	153	4.56	1.137
Communication	153	4.04	0.836
Senior management support	153	3.75	0.89
Time pressure	149	4.04	1.414
Job control	149	4.89	1.245
Cognitive demands	149	5.85	0.875
Safety climate	151	5.10	1.09
Job satisfaction	148	2.14	0.87
Job burnout	148	3.08	1.04

Table 3 shows the correlations between the two main outcome measures.

**Table 3:** Correlations between organisational variables and the two main outcome measures

	Job Satisfaction	Burnout
Organizational support	0.58***	-0.30***
Communication	0.51***	-0.33***
Senior management support	0.46***	-0.17*
Time pressure	-0.29***	0.49***
Job control	0.55***	-0.25**
Cognitive demands	0.01	0.28**
Hospital safety climate	0.46***	-0.27**
Age	0.08	0.16
Years of experience	0.06	0.15

*p < .05 ** *p* < .01 ***p < .001

There were significant bivariate correlations between job satisfaction and all predictor variables except cognitive demands, age and years of experience. All are positively associated with job satisfaction except time pressure as would be expected. For job burnout all seven organizational predictors were significantly correlated in the expected directions. Age and years of experience were not significantly correlated with burnout.

### Job satisfaction

Most of the participating ICPs reported being ‘just about satisfied’ (28 %, *n* = 42), ‘quite satisfied’ (24 %, *n* = 36) or ‘very satisfied’ (36 %, *n* = 54) with their work. With respect to predicting the overall job satisfaction of the ICPs, the regression model was significant with the combination of all nine variables able to explain 50 % of the variance in job satisfaction, R = .71 (R^2^ = 0.50), F (7,140) = 20.37, p < .001. Three variables of communication, job control and POS are significantly related to job satisfaction for ICPs (Table 4). The other variables are not significant predictors of job satisfaction.

**Table 4:** Regression model predicting job satisfaction in infection control practitioners

Variable	Beta	T
Perceived organizational support	0.229*	2.53
Communication	0.223*	2.23
Senior management support	0.122	0.44
Time pressure	-0.123	-1.85
Job control	0.363***	5.38
Cognitive demands	0.09	-1.19
Safety climate	-0.001	0.003

*p < .05; ***p < .001

### Emotional burnout

Most of the participating ICPs (60 %, *n* = 89*)* reported being burned out ‘occasionally’ (mean 2.14[1–5], SD 0.87). With respect to predicting the overall job burnout of ICPs, the regression model was significant with the combination of all nine variables able to explain 37 % of the variance in job satisfaction, R = .61 (R^2^ = 0.37), F(9,137) = 8.92,p < .001. High time pressure and cognitive demands coupled with poor communication are significantly related to high emotional burnout for ICPs (Table 5). The other variables are not significant predictors of emotional burnout.

**Table 5:** Regression model predicting job burnout in infection control practitioners

Variable	Beta	T
Perceived org support	-0.11	-1.08
Time pressure	0.33***	4.18
Job control	-0.14	-1.86
Communication	-0.26*	-2.31
Safety climate	-0.02	-0.15
Cognitive demands	0.17*	2.05
Support senior management	0.097	0.84
Age	0.23	1.54
Years of experience	0.1	-0.85

*p < .05; *** *p* < .001

## Discussion

In line with the first aim of our study we document the perceptions of the working conditions for ICPs and highlight the organizational context of ICPs in Australia. Our results suggest that whilst most ICPs perceive a good safety climate in relation to infection prevention and control practices in their organization, ICPs’ perceptions of organization support and support from senior management are moderate and variable, most are under significant time pressure and most experience high cognitive demands. Job satisfaction is moderate, although variable but is not related to age or to years of experience in the job.

With respect to the second aim, we show that low job satisfaction is linked to the organizational factors of poor communication, low job control and low POS. In contrast, emotional burnout is associated with organizational factors of high time pressure and cognitive demands coupled with poor communication.

In providing this evidence on the working conditions of ICPs in Australia, this study has made a significant contribution to the literature. Although there is some evidence on the scope of practice of ICPs in Australia [1, 2], there is no literature on the organizational context in which ICPs practice, nor how contextual factors affect ICPs’ practice. With the growing role and recognition of ICPs in the Australian context, these findings help add to the body or work demonstrating the need to define the scope of the ICP role and responsibilities [1, 2].

Job satisfaction among ICPs is independently predicted by low job control, low POS and poor communication about infection prevention and control practice. Job burnout among ICPs is predicted by high time pressure and cognitive demands coupled with poor communication. Organizational support, which [4] and [14] suggest is a key factor in predicting nurses’ experience of job dissatisfaction and burnout, predicted job satisfaction but *not* burnout. Conversely, time pressure was a significant predictor of burnout but not job satisfaction. Job control and communication predicted both job dissatisfaction and burnout. These results contribute to the understanding of the impact of key organizational factors on ICPs’ job experience. These findings are concordant with those in the nursing literature [3, 4] and have implications for the quality of ICPs’ infection prevention practices and, potentially, rates of HAIs in healthcare facilities.

The most significant organizational factors for both satisfaction and burnout are job control and communication. The literature suggests there are explanations for these relationships. Nurses, regardless of the context in which they practice, have a demanding work environment. However, where they perceive they have a degree of control over this environment, nurses can approach the difficulties it presents in a more positive way. Therefore, they experience a higher degree of personal accomplishment, which has been shown to be a key factor in job satisfaction and burnout [35]. Communication may impact on job satisfaction and burnout in a similar way, as communication is fundamental in enabling nurses to respond effectively to the challenges in their environment and, therefore, in promoting their sense of personal accomplishment [36]. Although there is no literature relevant to ICPs specifically, these findings can be feasibly extrapolated from the broader nursing to the specific ICP context.

It is interesting to note that although our study suggests job control and communication are predictive of job satisfaction and burnout among ICPs, the participating ICPs report both moderately high job control and very good communication practices in their organizations. Indeed, this could demonstrate a survival and selection effect such that only the ICPs that are able to work with control continue in these positions as well as them being the ones most likely to answer the survey.

On one of the strengths of this study was the high response rate with representation for all participating hospitals in each Australian state and territory, improving the generalizability of our findings and provided us with more confidence that a range of views and conditions have been captured. However, there were also some limitations. The sample size for this study whilst adequate for the analyses performed does not preclude the fact that some of the findings, particularly the smaller p values, may be attributable to chance.

The findings for aim 2 on the organizational factors which predict job dissatisfaction and burnout among ICPs cannot be linked to outcome measures – such as rates of HAIs. Although data on rates of HAIs in the participating hospitals is available from our concurrent evaluation of the NHHI, and the literature suggests there is indeed a relationship between organizational factors, stress / burnout and HAIs [11, 12, 37] due to the number of confounding variables involved in this study, identifying clear relationships between our predictor and outcome variables is problematic. It could be the case that cognitive demands drive burnout but equally it could be the other way around. The direction of the relationship is not able to be ascertained in this study.

We also acknowledge that the construct of job burnout was measured by a single item that has been validated against the emotional aspects of burnout as opposed to other aspects of burnout (like physical) so the relationships described here may not hold for other dimensions of burnout.

Additionally, due to ethical limitations related to data confidentiality, no hospital-level comparisons between the outcome measures could be undertaken. Finally, another key limitation to this study, which provides scope for further research, is that these findings only apply to large tertiary public hospitals in Australia; we cannot generalize these findings to the private hospital sector nor can we say whether and how these same organizational factors are relevant in remote or rural hospitals.

These findings may be used to modify national infection prevention and control programs to suit local organizational contexts, to help retain ICP staff and to improve ongoing training. These findings are also important in improving the effectiveness of infection control programs implemented in healthcare facilities – including in terms of promoting ICPs’ compliance with infection control practices and, potentially, achieving reductions in the risk of HAIs.

## Conclusions

Understanding the working conditions of ICPs is fundamental to a successful hospital infection prevention and control program. This study has provided evidence on the perceived working conditions of ICPS and discussed the associations between several organizational factors and job satisfaction and emotional burnout. Although this study cannot ascertain which organizational factors among ICPs cannot be linked to outcome measures (e.g. rates of HAIs), the findings presented are supported by data from a large number of ICPs in all Australian states and territories, and therefore represent a good cross-section of the Australian ICP population.

Overall, these findings represent an important contribution to understanding the working condition of specialized nursing group in Australia – one which is particularly relevant in the current climate of a global pandemic.

## Supplementary Information

Additional file 1

## Data Availability

The datasets used and/or analysed during the current study are available from the corresponding author on reasonable request.

## Abbreviations

ANOVA: Analysis of variances
HAIs: Hospital acquired infections
ICPs: Infection control practitioners
NHHI: National Hand Hygiene Initiative
POS: Perceived organizational support
