# Supplementary material for: A cross sectional study of organizational factors and their impact on job satisfaction and emotional burnout in a group of Australian nurses: infection control practitioners
Source: BMC Health Serv Res. 2021 May 10;21:441. doi: 10.1186/s12913-021-06477-2 (PMC8108460; doi:10.1186/s12913-021-06477-2)
Supplement: Supplementary file 1 — Additional file 1 [file 12913_2021_6477_MOESM1_ESM.pdf]

Support for Hand Hygiene

This questionnaire is designed to measure your opinions and attitudes about hand hygiene practices and the support they receive in your hospital.

Demographics

1. Are you:

Please pick one of the answers below.

- O Male
- O Female

2. How old are you?

Please use the blank space to write your answers.

....

3. Occupation

Please pick one of the answers below or add your own.

- O Enrolled nurse.
- O Registered nurse.
- O Clinical nurse.
- O Assistant in nursing.

Other

....

4. How many years experience do you have in nursing (including onsite training)?

(Please type a number in the box below)

Please use the blank space to write your answers.

....

5. What is your highest qualification?

Please use the blank space to write your answers.

....

6. What state is your hospital in?

Please pick one of the answers below.

- ○ Queensland
- ○ Victoria
- ○ Western Australia
- ○ South Australia
- ○ New South Wales
- ○ Northern Territory
- ○ Tasmania

7. What is the name of your hospital?

Please pick one of the answers below.

- ○ Royal Brisbane and Women's Hospital
- ○ Princess Alexandra Hospital
- ○ Gold Coast Hospital
- ○ The Prince Charles Hospital
- ○ Townsville Hospital
- ○ Cairns Base Hospital
- ○ Nambour Hospital
- ○ Ipswich Hospital
- ○ Logan Hospital

8. What is the name of your hospital?

Please pick one of the answers below.

- ○ Royal Adelaide Hospital
- ○ Flinders Medical Centre
- ○ The Queen Elizabeth Hospital Campus
- ○ Lyell-McEwin Hospital
- ○ Repatriation General Hospital

9. What is the name of your hospital?

Please pick one of the answers below.

- ○ Westmead Hospital
- ○ John Hunter Hospital
- ○ Royal Prince Alfred Hospital
- ○ Liverpool Hospital
- ○ Royal North Shore Hospital
- ○ St George Hospital
- ○ Prince Of Wales Hospital
- ○ Gosford Hospital
- ○ Nepean Hospital
- ○ Concord Hospital
- ○ Wollongong Hospital
- ○ Bankstown/ Lidcombe Hospital
- ○ St Vincent's Darlinghurst Hospital
- ○ Campbelltown Hospital
- ○ Blacktown Hospital

10. What is the name of your hospital?

Please pick one of the answers below.

- ○ Royal Hobart Hospital [including PICU]
- ○ Launceston General Hospital
- ○ NWRH Burnie

11. What is the name of your hospital?

Please pick one of the answers below.

- O Austin Hospital
- O The Alfred
- O Monash Medical Centre
- O Royal Melbourne Hospital
- O St Vincent's Hospital
- O Dandenong Campus
- O Geelong Hospital
- O Box Hill Hospital
- O Frankston Hospital
- O The Northern Hospital
- O Sunshine Hospital

12. What is the name of your hospital?

Please pick one of the answers below.

- O Royal Perth Hospital Wellington Street Campus
- O Sir Charles Gairdner Hospital
- O Fremantle Hospital
- O Princess Margaret Hospital
- O King Edward Memorial Hospital for Women

13. What is the name of your hospital?

Please pick one of the answers below or add your own.

- O Royal Darwin Hospital

Other

....

14. Have you worked at your current hosital for more than 6 months?

Please pick one of the answers below.

- O Yes
- O No

15. How long have you been working at your current hospital (years)?

Please write a number in the box below.

Please use the blank space to write your answers.

....

16. In which area do you work?

Please pick one of the answers below or add your own.

- ○ Infection Control
- ○ Safety and Quality

....

17. How long have you been working in infection control/safety and quality (years)?

Please write a number in the box below.

Please use the blank space to write your answers.

....

18. Do you supervise any staff in your current job?

Please pick one of the answers below and add your comments.

- ○ Yes
- ○ No

If Yes, how many?

....

19. Are you a hand hygiene auditor?

Please pick one of the answers below.

- ○ Yes
- ○ No

20. Are you a Gold Standard hand hygiene auditor?

Please pick one of the answers below.

- ○ Yes
- ○ No

21. To the best of your knowledge when was the "5 moments" program introduced at your hospital? (please give month and year)

Please use the blank space to write your answers.

....

22. Please indicate whether any of these changes have occured in your hospital since the introduction of the 5 moments for hand hygiene program and how (if at all) they affected the 5 moment program.

Please fill in the answers in the table below (mark appropriate circles and squares and fill in the blank spaces).

	Has this change occurred in your hospital?	Has this change affected the hand hygiene program?	How has it affected the program?	When did this change occur?
Change of district CEO	○ Yes<br>○ No	○ Yes<br>○ No	○ Generally positive effects<br>○ Generally negative effects	....
Change of hospital CEO	○ Yes<br>○ No	○ Yes<br>○ No	○ Generally positive effects<br>○ Generally negative effects	....
Change of health service district boundaries	○ Yes<br>○ No	○ Yes<br>○ No	○ Generally positive effects<br>○ Generally negative effects	....
Change in the manager of infection control/safety and quality	○ Yes<br>○ No	○ Yes<br>○ No	○ Generally positive effects<br>○ Generally negative effects	....
Change of infection control/safety and quality personnel	○ Yes<br>○ No	○ Yes<br>○ No	○ Generally positive effects<br>○ Generally negative effects	....
Significant change in infection control funding	○ Yes<br>○ No	○ Yes<br>○ No	○ Generally positive effects<br>○ Generally negative effects	....
Other 1 (please specify the type of change in the comments section below)	○ Yes<br>○ No	○ Yes<br>○ No	○ Generally positive effects<br>○ Generally negative effects	....
Other 2 (please specify the type of change in the comments section below)	○ Yes<br>○ No	○ Yes<br>○ No	○ Generally positive effects<br>○ Generally negative effects	....

23. Please describe any other significant changes (indicated above) that have occured in your hospital since the introduction of the 5 moments for hand hygiene program.

Please write your answer in the space below.

....

....

....

....

24. Please comment further on any of the above changes and their effects in more detail.

Please write your answer in the space below.

....

....

....

....

25. Please rate the extent to which you agree or disagree with the following statements.

Please mark the corresponding circle - only one per line.

	Strongly Disagree	Disagree	Somewhat Disagree	Neither Agree Nor Disagree	Somewhat Agree	Agree	Strongly Agree
My hospital cares about my opinions.	○	○	○	○	○	○	○
My hospital really cares about my well-being.	○	○	○	○	○	○	○
My hospital strongly considers my goals and values.	○	○	○	○	○	○	○
Help is available from my hospital when I have a problem.	○	○	○	○	○	○	○
My hospital would forgive an honest mistake on my part.	○	○	○	○	○	○	○
If given the opportunity my hospital would take advantage of me.	○	○	○	○	○	○	○
My hospital shows very little concern for me.	○	○	○	○	○	○	○
My hospital is willing to help me if I need a special favour.	○	○	○	○	○	○	○

26. SENIOR MANAGEMENT in your hospital

Please rate the extent to which you agree or disagree with the following statements:

Please mark the corresponding circle - only one per line.

	Strongly Disagree	Slightly Disagree	Neutral	Slightly Agree	Strongly Agree
Senior management has a clear picture of the risk associated with poor hand hygiene.	○	○	○	○	○
Senior management has a good idea of the actual hand hygiene mistakes in this hospital.	○	○	○	○	○
Hand hygiene decisions are made at the proper level by the most qualified people.	○	○	○	○	○
Senior management provides a climate that promotes hand hygiene.	○	○	○	○	○
Senior management considers hand hygiene issues when program changes are discussed.	○	○	○	○	○
My suggestions about hand hygiene would be acted upon if I expressed them to senior management.	○	○	○	○	○
Senior management does not knowingly compromise hand hygiene compliance for efficiency.	○	○	○	○	○
Senior management is driving us to be a hand hygiene centred hospital.	○	○	○	○	○

27. Please rate the extent to which you agree or disagree with the following statements:

Please mark the corresponding circle - only one per line.

	Strongly Disagree	Slightly Disagree	Neutral	Slightly Agree	Strongly Agree
**This hospital is doing more for hand hygiene that it did before "The 5 Moments" of hand hygiene was implemented.**	○	○	○	○	○
**Hand hygiene has become a major area for improvement in this hospital.**	○	○	○	○	○

28. Communication about Hand Hygiene

Please mark the corresponding circle - only one per line.

	Strongly Disagree	Slightly Disagree	Neutral	Slightly Agree	Strongly Agree
**I know the proper channels to direct questions regarding hand hygiene.**	○	○	○	○	○
**Good communication flow exists up the chain of command regarding hand hygiene.**	○	○	○	○	○
**Good communication flow exists down the chain of command regarding hand hygiene.**	○	○	○	○	○
**Senior management is successful in communicating its hand hygiene goals to employees.**	○	○	○	○	○

29. Please rate the extent to which you agree or disagree with the following statements.

MY HOSPITAL:

Please mark the corresponding circle - only one per line.

	Strongly Disagree	Disagree	Somewhat Disagree	Neither Agree Nor Disagree	Somewhat Agree	Agree	Strongly Agree
Reacts quickly to solve the problem when told about infection-related risks	○	○	○	○	○	○	○
Insists on regular and thorough hand hygiene audits	○	○	○	○	○	○	○
Tries to continually improve hand hygiene compliance in each ward	○	○	○	○	○	○	○
Provides all the products and equipment needed to engage in hand hygiene	○	○	○	○	○	○	○
Is strict about maintaining hand hygiene when work falls behind schedule	○	○	○	○	○	○	○
Quickly corrects any infection-related hazard (even if it is costly)	○	○	○	○	○	○	○
Provides detailed hand hygiene compliance reports to employees	○	○	○	○	○	○	○
Considers a person's hand hygiene compliance when rewarding staff	○	○	○	○	○	○	○
Requires each nurse unit manager to help improve hand hygiene in his/her department	○	○	○	○	○	○	○
Invests a lot of time and money in hand hygiene training for employees	○	○	○	○	○	○	○
Uses any available information to improve existing hand hygiene rules	○	○	○	○	○	○	○

Listens carefully to employees' ideas about improving hand hygiene	○	○	○	○	○	○	○
Considers hand hygiene when setting workload and schedules	○	○	○	○	○	○	○
Provides health care workers with a lot of information on hand hygiene issues	○	○	○	○	○	○	○
Regularly holds hand hygiene awareness events (e.g., presentations, ceremonies)	○	○	○	○	○	○	○
Gives infection control personnel the power they need to do their job	○	○	○	○	○	○	○

30. Please rate your response to the following statements.

To what extent:

Please mark the corresponding circle - only one per line.

	Never						Always
Does your work need your undivided attention?	○	○	○	○	○	○	○
Do you have to keep track of more than one process at a time?	○	○	○	○	○	○	○
Do you have to concentrate all the time to watch for things going wrong?	○	○	○	○	○	○	○
Do you have to react quickly to prevent problems arising?	○	○	○	○	○	○	○

31. Please rate your response to the following statements.

Please mark the corresponding circle - only one per line.

	Never						Always
I have unachievable deadlines.	O	O	O	O	O	O	O
I have to neglect some tasks because I have to much to do.	O	O	O	O	O	O	O
I have unrealistic time pressures.	O	O	O	O	O	O	O
I have a say in my own work speed.	O	O	O	O	O	O	O
I have a choice on deciding what I do at work.	O	O	O	O	O	O	O
I have a choice in how I do my work.	O	O	O	O	O	O	O

32. Please choose one statement that most accurately describes your circumstances at work.

Please pick one of the answers below.

- O I enjoy my work. I have no symptoms of burnout.
- O Occasionally I am under stress, and I don't always have as much energy as I once did, but I don't feel burned out.
- O I am definitely burning out and have one or more symptoms of burnout, such as physical and emotional exhaustion.
- O The symmptoms of burnout that I'm experiencing won't go away. I think about frustration at work a lot.
- O I feel completely burned out and often wonder if I can go on. I am at the point where I may need some changes or may need to seek some sort of help.

33. Please rate your response to the following question:

Please mark the corresponding circle - only one per line.

	Not at all satisfied	Just about satisfied	Quite satisfied	Very satisfied	Extremely satisfied, couldn't be more satisfied.
How do you feel about your job, all things considered?	O	O	O	O	O

34. Do you have any other comments you wish to make about the "5 moments" hand hygiene program in your hospital?

Please write your answer in the space below.

....

....

....

....

Review Your Responses
